# Long-term follow-up of bone density changes in total hip arthroplasty: comparative analysis from a randomized controlled trial of a porous titanium construct shell vs. a porous coated shell

**DOI:** 10.1007/s00264-024-06289-z

**Published:** 2024-09-07

**Authors:** Michael Axenhus, Mats Salemyr, Sebastian Mukka, Martin Magnéli, Olof Sköldenberg

**Affiliations:** 1 Division of Orthopaedics, Department of Clinical Sciences at Danderyd Hospital, Karolinska Institutet, Entrévägen 2, 182 88, Danderyd, Stockholm, Sweden; 2 Department of Diagnostics and Intervention (Orthopaedics), Umeå University, Umeå, Sweden

**Keywords:** Bone mineral density, Implant material selection, Long-term follow-up, Periacetabular bone loss, Porous titanium cup, THA

## Abstract

**Purpose:**

Periacetabular bone loss poses a considerable challenge in the longevity and stability of acetabular implants used in total hip arthroplasty (THA). Innovations in implant design, specifically the introduction of three-dimensional (3D) porous titanium constructs, might reduce bone resorption. The purpose of this study was to build upon our previous randomized controlled trial, which found no change in periacetabular bone loss between a 3D porous none-hydroxyapatite coated titanium cup and a standard porous hydroxyapatite coated cup over a two year follow-up period by extending the follow-up duration to ten years post-surgery.

**Methods:**

This was a single-centre, long-term follow-up study conducted over a ten year period in patients who had previously participated in a randomized controlled trial comparing a 3D porous titanium construct shell (PTC group) with a standard porous hydroxyapatite coated titanium shell (PC-group). The primary outcome measured was the change in bone mineral density (BMD) within four specific periacetabular zones, alongside overall bone loss, which was assessed through BMD in the lumbar spine at two, six and ten years postoperatively. Secondary outcomes included clinical outcome measures.

**Results:**

In total, 18 in the PTC and 20 in the PC group were analysed for the primary endpoint up to ten years. The mean bone mineral density in zones 1–4 was 3.7% higher in the PTC group than in the PC group at six years postoperatively and 12.0% higher at ten years. Clinical outcomes, and the frequency of adverse events did not differ between the groups.

**Conclusions:**

The PTC group displayed superior long-term bone preservation compared to the PC group while maintaining similar clinical outcomes up to ten years postoperatively. Although with a small sample size, our findings suggest that porous titanium cups have the potential to minimize BMD loss around the cup which could contribute to improving THA outcomes and implant durability.

**Supplementary Information:**

The online version contains supplementary material available at 10.1007/s00264-024-06289-z.

## Introduction

Periacetabular bone loss represents a significant concern in the context of long-term stability and survivorship of acetabular implants utilized in total hip arthroplasty (THA) [1–3]. Factors such as periacetabular adaptive bone remodeling, commonly known as stress shielding, along with osteolysis induced by wear debris, exert substantial influence on periacetabular bone mass. Advancements in implant design have introduced three-dimensional (3D) porous titanium constructs integrated into acetabular shells, have been suggested to mitigate adaptive bone resorption and facilitate osseointegration in THA [4]. However, empirical evidence of the in-vivo effects of these innovations on human subjects remains limited [5]. The aim of this study was to serve as a long-term follow-up study building upon a randomized controlled trial comparing the performance of a 3D porous titanium construct shell (PTC) against a standard porous hydroxyapatite coated titanium shell (PC) at preventing bone mineral loss [6]. Our specific research questions were: how does PTC change periacetabular bone mineral density (BMD) during the ten years follow up? How does PTC and PC differ in terms of clinical outcome and complications?

## Methods

### Trial design

This study is a follow-up study of a single-centre randomized clinical trial [6]. The original trial aimed to investigate and compare the long-term effects of distinct acetabular implants used in THA on periacetabular bone density changes and clinical outcomes. Participants meeting inclusion criteria were recruited within a designated time frame (October 2009 to August 2013) at the Orthopaedic Department of Danderyd Hospital, in collaboration with the Department of Clinical Sciences at Karolinska Institute in Stockholm. The CONSORT statements were followed [7].

### Participant selection and surgical procedure

We included individuals diagnosed with primary osteoarthritis, aged between 40 and 70 years, who were eligible for uncemented cups without significant structural acetabular defects and a femur type A or B according to Dorr [8]. Exclusions included prior hip surgeries on the affected side and regular use of corticosteroids, bisphosphonates or cytostatic drugs. A BMI of 35 or more was also set as an exclusion criterion. Patients provided informed consent before participation.

Patients allocated to the PTC group received an acetabular shell characterized by a 3D-printed porous titanium backside surface (Regenerex™-shell, E1™-liner, Biomet, USA) [9]. This implant featured a 1.5-mm thick trabecular-like porous titanium structure on its backside, with a porosity of 67% and an average pore size of 300 μm. The PC group underwent surgery with a titanium shell with hydroxyapatite (Pinnacle Duofix™-shell, Marathon™-liner, Depuy Johnson & Johnson, USA) [10], which comprised a porous coating composed of sintered titanium beads with an average pore size of 250 μm. Both liners utilized highly cross-linked polyethylene (HXLPE). On the femoral side, a 32-mm cobalt-chrome head was utilized alongside uncemented implants from either of the manufacturers (Bimetric™, Biomet, USA and Proxima™, Depuy Johnson & Johnson, USA). A posterolateral approach were used and all surgeries were performed by one of five experienced senior hip surgeons [11]. Postoperatively, patients were encouraged to use crutches according to individual preference during the initial weeks. Full weight-bearing was permitted as tolerated. Rehabilitation was supervised by a physiotherapist both during the hospital stay and subsequently in a day-clinic setting for the initial few weeks.

### Endpoints and evaluation over extended intervals

The primary endpoint was change in BMD at the 6- and 10-year mark, with secondary endpoints focusing on functional clinical results. Follow-ups were scheduled at two, six and ten years postoperatively. All data was collected by research nurses who also acted as study coordinators. All data was collected and kept in Research Electronic data capture (REDCap), a digital case report form [12].

### Bone densitometry

**Fig. 1:**
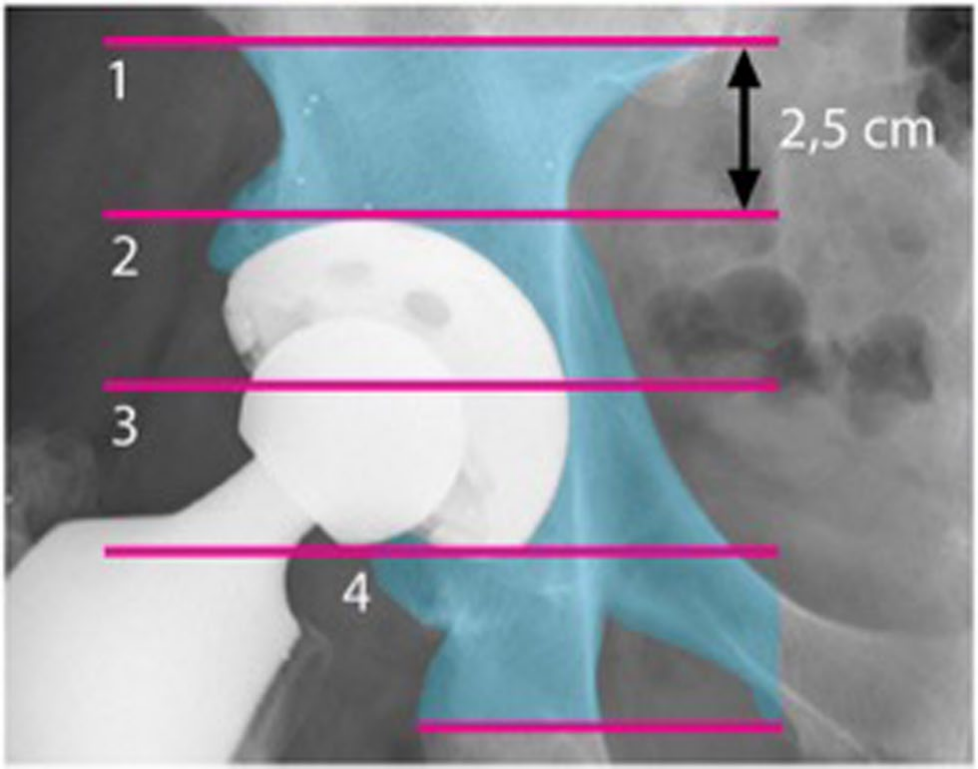
Bone mineral density measurement zones

BMD within four specific zones, as outlined by Wilkinson et al. [13] and subsequently refined by Laursen et al. [14] (Fig. 1), was assessed in this study. The change in BMD within each zone was calculated by comparing the measured BMD values during follow-up examinations with the baseline value obtained two days after the surgery. This change was expressed as a percentage relative to the baseline measurement. To ensure consistency and accuracy, individual regions of interest (ROI) for each patient were saved and utilized for subsequent examinations, minimizing measurement errors.

BMD measurements from the lumbar spine at the level of L1-4 were also collected at the same time points as described above in order to assess overall bone loss and compare overall bone loss to BMD changes in the cup area. Only patients with complete BMD datasets for all follow ups were analyzed.

DXA scanning was conducted using a General Electric Healthcare machine, specifically the Lunar Prodigy Advance (GE Healthcare, Pittsburgh, US), operated with version 13.31.016 software. Rigorous quality controls of the DXA equipment were performed in adherence to the manufacturer’s guidelines throughout the study period, and no deviations from optimal functionality were identified.

### Clinical outcome assessment

Clinical evaluations consisted of two self-administered scoring protocols, Harris hip score [15] and EQ-5D [16]. These were administered at each follow-up visits. Reoperation rate was followed by a review of the medical records and by asking patients at follow-up meetings whether they had gone through any revision surgery.

### Statistical analysis

Emphasizing the significance of ROI 1 and 2, situated proximal to the cup, we considered compromised bone stock in these zones critical in the context of potential future cup revisions. We hypothesized that a mean difference of 10% with a standard deviation of 10% in bone mineral density within ROI 1 and 2 represented the smallest clinically relevant difference [13, 17]. Subjects with missing BMD data during follow-up visits were managed by employing the last observation carried forward method. This approach was used for individual follow-ups in both the PTC and PC groups. The study was analyzed according to the intention-to-treat principle. Given the normal distribution of both datasets, between-group comparisons were conducted using the unpaired Student t-test. All statistical analyses were performed using SPSS 22.0.

#### Ethics and registration

Regional ethics committee approval (number 2008/4:3) and ClinicalTrials.gov registration (number NCT01319227) was obtained to ensure adherence to ethical standards and transparent trial documentation.

### Participant flow and baseline data

**Fig. 2:**
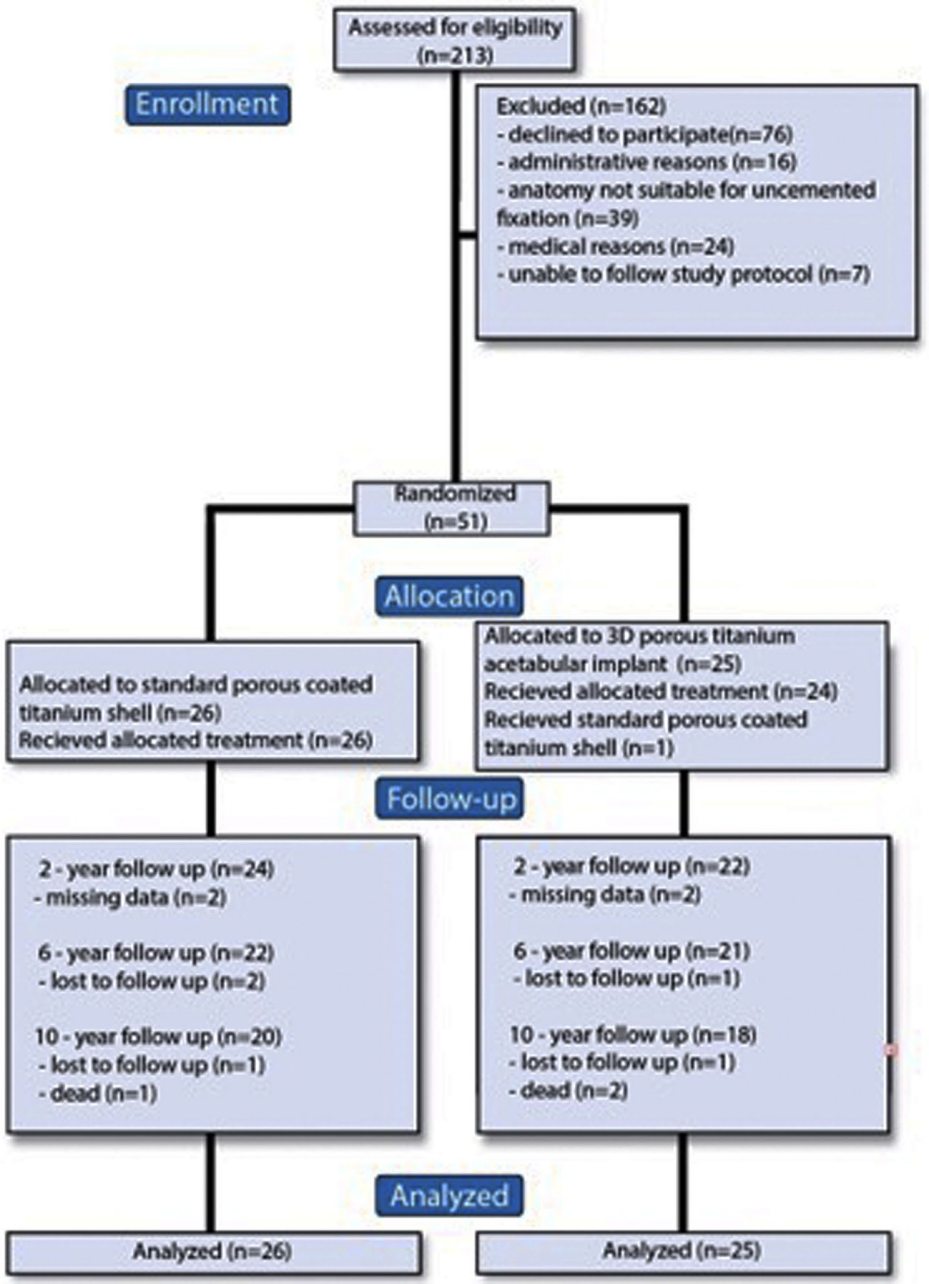
CONSORT flow diagram

A total of 51 patients were enrolled (22 males and 29 females), mean age was 62 years. The baseline characteristics of both groups were similar. There was one death as a participant passed away from unrelated reasons five months after inclusion. 43 patients remained at the six years follow up and 38 at the ten year follow up (Table 1) (Fig. 2).

**Table 1:** Baseline characteristics of study participants. ASA: American Society of anesthesiologists physical status classification system, BMD: periacetabular bone mineral density, BMI: body mazz index, WOMAC: Western Ontario and McMaster universities osteoarthritis Index

Baseline Characteristics	Standard porous coated titanium shell (*n* = 26)	3D porous titanium aetabular implant (*n* = 25)
Age, yearsa	62 ± 5	62 ± 6
Male/ Female, n	11/15	11/14
Weight, kga	79 ± 15	82 ± 13
Height, cma	171 ± 10	172 ± 8
BMIa	27 ± 4	28 ± 4
Charnley class, n (A/ B/ C)	16/ 10/0	18/ 7 /0
ASA Class, n (1–2 / 3–4)	21/5	20/5
Harris hip score, preop ^0^	56 (29–68)	46 (10–70)
WOMAC score preop ^0^	45 (15–70)	43 (S-72)
Normal bone density	13	12
Osteopenia	8	7
Osteoporosis	2	0
BMD “,g/cm2	1.14 ± 0.21	1.15 ± 0.18
Surgery, n		
Cup, (Pinnacle/ Regenerex)	26/ 0	1/24
Articulation, mm (32 / 28)	26/0	24/1

a = mean(SD)

0 = median(range)

## Results

### Primary endpoint

**Fig. 3:**
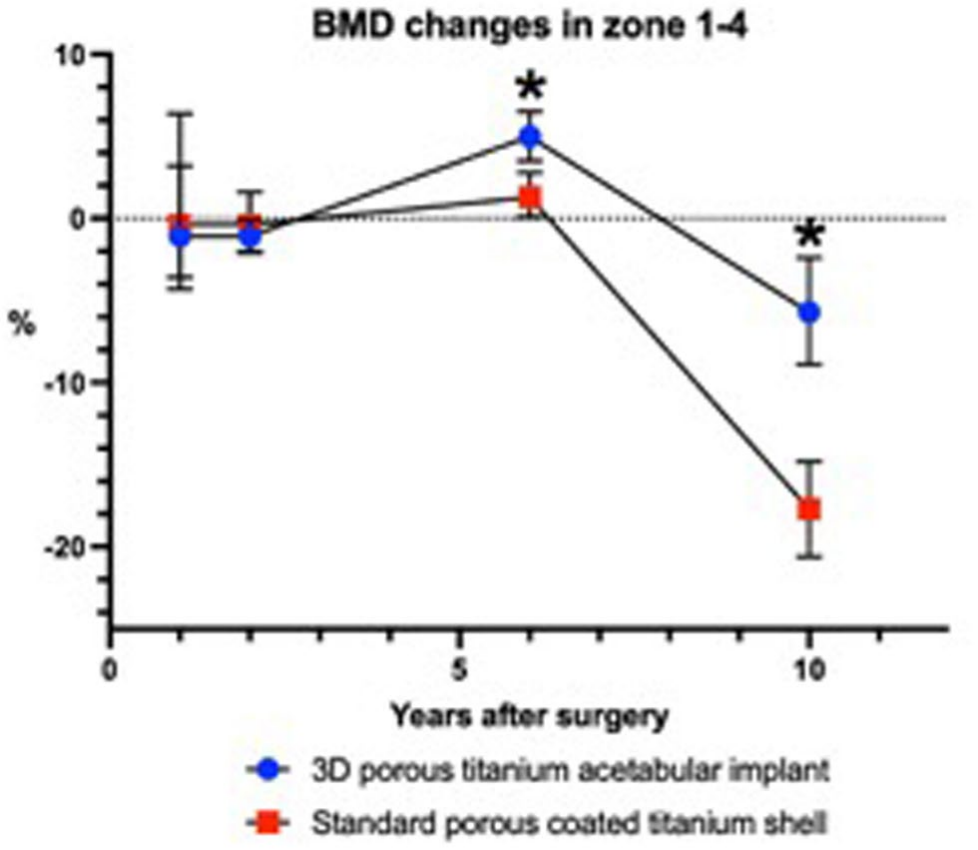
Periacetabular bone mineral density (BMD) change (%) in zone 1–4. Error bars indicate 95% confidence interval. Significance indicate difference between cup groups (*p* < 0.05)

At the 6-year follow-up the PTC group had a substantial increase (1.3%±1.4) in BMD. At the ten year follow-up the PTC group experienced a smaller reduction in BMD (-5.7% ±3.3) compared to the PC group (-17.7% ±2.9), (Table 2) (Fig. 3).

**Table 2:** Primary endpoint, bone remodeling measured by dual energy X-ray absorptiometry, expressed as change in periacetabular bone mineral density (%)

Outcome	Standard porous coated titanium shell (*n* = 20)	3D porous titanium acetabular implant (*n* = 18)	Difference in % (95% CI)	*p*-value
Change in BMD Zone 1				
2 years	-2.1 ± 6.6	-6.0 ± 6.7	-3.9 (-0.2 to 7.7)	0.042
6 years	-0.01 ± 7.1	-7.3 ± 6.5	-7.3 (-10.6 to -4.1)	0.036
10 years	-21.7 ± 8.8	-6.0 ± 10.1	14.9 (5.1 to 24.8)	**< 0.001**
Change in BMD Zone 2				
2 years	-10.3 ± 9.6	-4.4 ± 20.0	6.0 (-15.1 to 3.2)	0.192
6 years	-11.3 ± 6.1	11.2 ± 6.3	-0.1 (-2.9 to 3.2)	0.615
10 years	-29.7 ± 8.1	-17.8 ± 8.6	11.9 (3.6 to 20.2)	0.040
Change in BMD Zone 3				
2 years	1.7 ± 14.7	7.0 ± 21.7	5.3 (-16.0 to 5.5)	0.323
6 years	3.6 ± 6.0	1.2 ± 5.3	-2.4 (-5.2 to 0.4)	0.126
10 years	-15.8 ± 7.9	4.3 ± 6.8	11.5 (3.7 to 19.3)	0.037
Change in BMD Zone 4				0.595
2 years	10.2 ± 16.8	7.8 ± 15.1	-2.4 (-6.7 to 11.5)	0.595
6 years	1.9 ± 5.5	1.6 ± 5.1	-0.3 (-2.9 to 2.6)	0.223
10 years	-4.1 ± 7.3	9.9 ± 7.3	-5.8 (-13.5 to 1.9)	0.187
Change in BMD Zone 1–4				
2 years	-0.4 ± 5.0	-1.1 ± 3.8	-1.5 (-2.8 to 5.9)	0.483
6 years	1.3 ± 1.4	5.0 ± 1.5	3.7 (1.4to 6.1)	0.028
10 years	-17.7 ± 2.9	-5.7 ± 3.3	12.0 (6.1 to 17.9)	**< 0.001**

The overall bone loss in the cohort was − 0.009 g/cm^2^ as measured by BMD in the lumbar spine, L1-4. The PTC group did not lose significantly more BMD around the cup when compared to overall bone loss. The PC group lost significantly more BMD around the cup compared to overall bone loss (*p* = 0.002) (Table 3).

**Table 3:** Difference in Periacetabular bone mineral density (BMD) loss in Zone 1–4 and in the lumbar spine L1-4. Values are given in g/cm^2^. Difference is represented as the change between loss in L1-4 versus zone 1–4

	BMD L1-4 (*n* = 51)	BMD zone 1–4 Standard porous coated titanium shell (*n* = 20)	BMD zone 1–4 3D porous titanium acetabular implant (*n* = 18)
Preop (SD)	1.162 ± 0.19	1.121 ± 0.26	1.113 ± 0.26
6 years (SD)	1.157 ± 0.21	1.108 ± 0.23	1.058 ± 0.24
10 years (SD)	1.152 ± 0.21	0.945 ± 0.27	1.055 ± 0.22
Preop compared to 10 years (SD)	-0.009 ± 0.20	-0.176 ± 0.24	-0.058 ± 0.24
p-value	0.846	0.0077	0.371
Difference(%) compared to L1-4 (SD)		-12.0 ± 2.6 -9.2 ± 3.1	
p-value 0.002 0.1824			

### Secondary endpoint

The preoperative assessments for Harris Hip Score and EQ-5D score showed no significant differences between PTC group and the PC group. At the six year follow-up, both groups had improvements in Harris Hip Score and EQ-5D score compared to baseline. At the ten year follow-up, both groups exhibited improved functional outcomes compared to baseline. The Harris Hip Score and EQ-5D score showed no significant variations between the PTC group and the PC group (Table 4).

**Table 4:** Clinical outcome at 6- and 10-years follow-up

	Standard porous coated titanium shell (*n* = 26)	3D porous titanium acetabular implant (*n* = 25)
Age	62 ± 5	62 ± 6
Male/ Female, n	11 / 15	11 / 14
Harris hip score, preop b	56 (29–68)	46 (10–70)
EQ-5D b	69 (64–74)	69 (66–72)
**6 year follow up**		
Male/ Female, n	11/11	11/10
Harris hip score b	89 (49–100)	92 (54–100)
EQ-5D b	81 (29–100)	84 (33–100)
**10 year follow up**		
Male/ Female, n	10 / 10	9/9
Harris hip score b	87 (63–100)	91 (51–100)
EQ-5D b	79 (48–100)	80 (48–100)

a = mean (SD)

b = median (range)

### Adverse events

The PTC group had one case of dislocation, two cases of periprosthetic joint infection and one case of trochanteritis. Reoperations in this group included stem revision, and debridement, antibiotics and implant retention (DAIR) procedures, each performed once. The total number of reoperations and complications was also two and four, respectively. In the PC group, adverse events included one case each of dislocation, excessive subsidence/loosening of the stem, new fracture distal to stem (Vancouver C) and back pain. Additionally, this group underwent reoperations comprising of a single ORIF and a single stem revision. The total count of reoperations was two, with a total of four reported complications (Supp. Table 1).

## Discussion

In this long-term follow-up of a randomized clinical trial, we found a lower decrease in periacetabular bone density in a none-hydroxyapatite coated PTC compared to hydroxyapatite coated PC. Our findings align with previous studies utilizing porous titanium acetabular cups which show favourable outcomes [18–21]. Our study is, to our knowledge, the only study that has examined long-term BMD changes and clinical outcomes of the use of PTC in THA.

### Primary endpoint

We found significant differences in periacetabular BMD alterations between the PTC group and the PC group. The PC group exhibited considerable declines in BMD over the ten year follow-up period compared to the PTC group. The PTC group also showed less BMD loss when comparing overall bone loss using lumbar spine measurements. Our findings can be compared to the results of Massari et al. who found that porous titanium cups displayed stable BMD up to two years post operatively [22]. In similarity, Sodhi et al. found no radiographic failures of highly porous acetabular cups in primary THA with a minimum follow up of two years and seven months [23]. Although small, our study suggest that the PTC shells has the potential to mitigate long-term acetabular bone loss more effectively compared to PC shells. There are patient groups that could potentially benefit from the use of porous titanium cups, as uncemented hydroxyapatite coated porous cups in female patients with low systemic bone mineral density have been shown to be prone to cup migration [24]. Bone ingrowth and implant fixation might be further improved by variations in technical specifications such as pore size [25].

### Secondary endpoint

Harris Hip Score and EQ-5D, revealed no significant differences between the PTC and PC groups at the six and ten year follow-ups. Both groups exhibited considerable improvements in functional outcomes compared to baseline.

### Bone remodeling

Periacetabular bone remodeling is a well-documented phenomenon in THA, often characterized by a reduction in BMD in proximal regions and occasional minor reductions or even increases in distal areas, as reported in previous studies [17, 22, 26–28]. We found that the porous titanium cup exhibited a noticeable preservation of bone in zones 3 and 4. This observation might be explained by the increased porosity and trabecular-like geometry of the coating in this specific cup, potentially facilitating a deeper and more uniformly distributed bone ingrowth compared to the surface of the PC group. These features could explain the observed trend of higher BMD values measured in the distal zones.

The potential for a more robust shell fixation in zones 3 and 4 of the PTC might have resulted in reduced compression forces transmitted to the proximal periacetabular bone, contributing to the comparatively larger reduction in BMD observed in zone 1 of the PTC group [29]. Moreover, the phenomenon of more pronounced bone preservation in the distal two zones than in the proximal two could be attributed to a well-fixed shell inducing traction forces acting on the periacetabular bone distally which could stimulate an increase in bone mineral density, possibly explaining the BMD retention observed in zones 3 and 4. The proximity of the implanted metal shell to the native bone underlying the acetabular fossa could also have altered the load pattern in this zone, influencing BMD. Overall, these findings align with our initial arguments, reinforcing the potential role of the porous titanium cup’s design features in encouraging enhanced osseointegration and bone-preserving, particularly in the distal periacetabular zones, through mechanisms such as improved bone ingrowth and altered load patterns.

### Adverse events

Both the PTC and PC groups exhibited similar adverse event rates and subsequent reoperations. Each group reported four adverse events, including issues like dislocation, subsidence/loosening of the stem, prosthesis joint infection, back pain, and trochanteritis. The comparable incidence of adverse events suggests a similar safety profile for both implant types, although the number of adverse events were few.

### Limitations

There are some limitations to our study. There was a relatively small sample size and there is a need for larger cohorts to validate our findings. Additionally, the evaluation focused on specific BMD changes and functional outcomes, necessitating a comprehensive assessment of implant longevity and survivorship. Furthermore, the presence of compromised bone stock can influence implant performance and outcomes in THA. While our cohort did not include individuals with deficient bone stock, it is important to note that implant selection in cases of poor bone quality may yield different results in terms of bone remodeling and clinical outcomes.

## Conclusion

In conclusion, our study suggests a role for PTC in preserving periacetabular bone density following THA. We found that the porous titanium cup without hydroxyapatite has advantages in mitigating long-term acetabular bone loss compared to a standard hydroxyapatite coated porous cup while maintaining clinical outcomes. Further research in larger cohorts with long-term follow-up is warranted to corroborate these findings.

## Electronic supplementary material

Below is the link to the electronic supplementary material.

Supplementary Material 1

## Data Availability

The datasets generated and analysed during the current study are available from the corresponding author on reasonable request.
