# Supplementary figures and images for: Long-term follow-up of bone density changes in total hip arthroplasty: comparative analysis from a randomized controlled trial of a porous titanium construct shell vs. a porous coated shell
Source: Int Orthop. 2024 Sep 7;48(11):2835–42. doi: 10.1007/s00264-024-06289-z (PMC11490515; doi:10.1007/s00264-024-06289-z)

Supplemental material


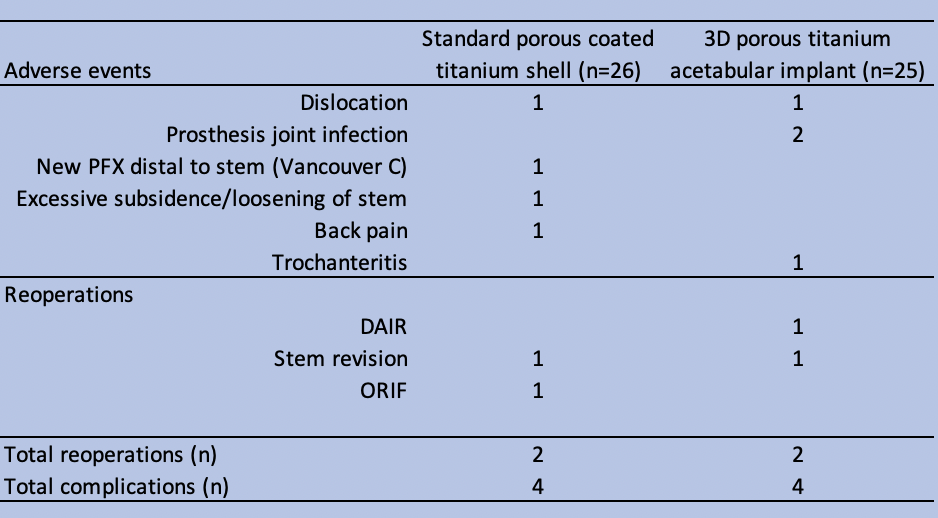


Supplemental table 1: Adverse events during the 10 years follow-up period.

Supplement: Supplementary file 1 — Supplementary Material 1 [file 264_2024_6289_MOESM1_ESM.docx]
